# Effect of a silicon carbide bur on shear bond strength between resin cement and zirconia

**DOI:** 10.2340/biid.v13.46767

**Published:** 2026-08-28

**Authors:** Khalil Aburawa, Kamel Al Hijazi, Christel Larsson, Martin Janda

**Affiliations:** a Department of Prosthodontics, Faculty of Odontology, Malmö University, Malmö, Sweden; b Department of Prosthetic Dentistry, Riga Stradins University, Riga, Latvia; c Chulalongkorn University Implant and Esthetic Center, Faculty of Dentistry, Chulalongkorn University, Bangkok, Thailand

**Keywords:** Zirconia, adhesive resin cement, silicon carbide bur, shear bond strength, airborne-particle abrasion

## Abstract

**Purpose:**

This in-vitro pilot study aimed to investigate the effect of a silicon carbide grinding bur on the shear bond strength (SBS) between zirconia and 10-MDP-containing cement.

**Materials and methods:**

Thirty zirconia specimens were prepared and randomly assigned to three groups based on surface pretreatment: airborne-particle abrasion with aluminum oxide particles (APA), treatment with a silicon carbide grinding bur (SiC bur), and airborne-particle abrasion followed by silicon carbide bur treatment (APA + SiC bur). All specimens were bonded with an adhesive resin cement (PANAVIA V5), aged with 5,000 thermocycles, and subjected to an SBS test. Statistical analysis was performed using one-way analysis of variance (ANOVA) and Tukey’s post hoc test (α = 0.05). Fracture modes were assessed.

**Results:**

The APA group showed significantly higher SBS than the SiC bur and APA + SiC bur groups (*p* < 0.001). The APA + SiC bur group showed a higher mean SBS than the SiC bur group, but the difference was not statistically significant (*p* > 0.05).

**Conclusion:**

Silicon carbide bur treatment did not have a beneficial effect on SBS after 5,000 thermocycles. Airborne-particle abrasion resulted in significantly higher SBS than silicon carbide bur treatment alone or airborne-particle abrasion followed by silicon carbide bur treatment.

**Clinical relevance:**

Under the tested conditions, the silicon carbide bur cannot be recommended as an alternative or an adjunct to airborne-particle abrasion for improving zirconia-resin cement bonding.

**KEY MESSAGES**

- Airborne-particle abrasion resulted in higher shear bond strength after 5,000 thermocycles than silicon carbide bur treatment alone or sequential airborne-particle abrasion and silicon carbide bur treatment.
- The silicon carbide bur did not provide a beneficial effect and cannot be recommended as an alternative or adjunct to airborne-particle abrasion under the tested conditions.
- Because untreated zirconia was not included, the effect of silicon carbide bur treatment relative to no surface treatment could not be determined.

## Introduction

Yttrium oxide-stabilized tetragonal zirconium dioxide polycrystals (Y-TZP), commonly referred to as zirconia, are oxide ceramics that lack a glass phase and have a crystalline structure. This crystalline structure imparts greater stability and strength compared to other glass-based ceramics. However, it may affect bonding between the tooth and the prosthetic restoration. Reliable bonding is a critical factor for achieving long-term success in prosthodontic treatment, particularly with the minimal invasive and adhesive approaches of modern dentistry. Surface treatment of prosthetic restorations is a well-established method to enhance the bond strength between the tooth and the restoration, thereby reducing the risk of loss of retention. This treatment is accomplished through chemically or mechanically modifying the bonding surface of the restoration. Oxide ceramics are more difficult to etch than porcelain or glass-ceramics. Etching oxide ceramics requires specific conditions, such as high acid concentration, prolonged exposure time, and high temperature [1]. Furthermore, because oxide ceramics lack a glass phase, etching does not yield the same effect as it does on porcelain or glass-ceramics [2]. Currently, airborne-particle abrasion with aluminum oxide particles is the most commonly used and most effective surface treatment method for zirconia [3]. This process increases surface roughness and surface energy, thereby enhancing micromechanical retention [4]. A notable drawback with particle abrasion is the inevitable formation of microdefects and sharp crack tips, which leads to potential material loss, particularly at the margin of the restoration, thereby compromising the mechanical strength of the material [3, 5]. The use of a primer or resin cement containing 10-MDP has been shown to enhance bonding to zirconia [6].

Many ceramic repair systems have been developed to provide more time- and cost-efficient treatment alternatives to avoid the full replacement of defective restorations. These systems aim to enable the direct intraoral repair of ceramic defects using a resin composite. Previous studies have shown that the Cimara Repair System (Cimara, VOCO GmbH, Cuxhaven, Germany) demonstrates high bond strength between repair materials and ceramic surfaces, including zirconia [7–9].

Airborne-particle abrasion with aluminum oxide particles, particularly when combined with an MDP-containing primer or resin cement, is an established method for improving resin bonding to zirconia [4, 6]. This treatment increases surface roughness, wettability, and surface energy, thereby promoting micromechanical retention. However, the resulting surface characteristics and bond strength depend on several treatment parameters, including particle size, pressure, distance, and application time [6, 10].

In a previous study comparing five ceramic repair systems, the VOCO repair system, which included a silicon carbide bur, produced unexpectedly high shear bond strength (SBS) to zirconia [9]. However, because the repair systems differed in several components and application procedures, the specific contribution of the silicon carbide bur could not be determined. This finding provided the rationale for the present study, which was designed to compare silicon carbide bur treatment with established airborne-particle abrasion and with their sequential combination.

Therefore, this in-vitro pilot study aimed to compare the SBS between zirconia and an MDP-containing resin cement following three surface-pretreatment protocols: airborne-particle abrasion, silicon carbide bur treatment, and sequential airborne-particle abrasion followed by silicon carbide bur treatment.

The null hypothesis was that there would be no significant difference in SBS after artificial aging among zirconia specimens treated with airborne-particle abrasion, a silicon carbide bur, or their sequential combination.

## Materials and methods

The sample-size calculation was based on the primary comparison between airborne-particle abrasion and silicon carbide bur treatment. The assumed within-group standard deviation of 7 MPa was based on the dispersion observed among the zirconia groups in a previous related in-vitro study that included the same silicon carbide bur, in which standard deviations of up to 6.3 MPa were reported [9]. A standard deviation of 7 MPa was therefore selected as a conservative estimate. Assuming a two-sided significance level of 5% (α = 0.05), a statistical power of 80%, and a predefined relevant difference of 10 MPa, corresponding to a standardized effect size of *d* = 1.43, the normal-approximation formula for two independent means indicated a minimum sample size of eight specimens per group. Ten specimens per group were included. The calculation was performed manually, without statistical software.

### Specimen preparation

Thirty (*n* = 30) zirconia specimens were manufactured from Copra Supreme Symphony zirconia (Whitepeaks Dental Solutions GmbH, Essen, Germany). Copra Supreme Symphony was selected as a commercially available zirconia routinely used by the collaborating dental laboratory at the time of the study. It is a precolored, polychromatic multilayer zirconia containing approximately 4 mol% yttria (4Y-PSZ) and was used in an unveneered, monolithic form. Each specimen consisted of two components: a block-shaped piece (7 × 7 × 14 mm) and a cylindrical piece (5 mm in diameter and 5 mm in height).

The specimens were designed using computer-aided design (CAD) software (AutoCAD, Autodesk, Inc., San Francisco, CA, USA). The design files were subsequently processed using computer-aided manufacturing (CAM) software (dentalcam) and machine-control software (dentalcnc; vhf camfacture AG, Ammerbuch, Germany).

After milling, the zirconia specimens were sintered in an HTS-2/M/Zirkon-120 furnace (MIHM-VOGT GmbH & Co. KG, Stutensee-Blankenloch, Germany) using the Translucency Program recommended by the zirconia manufacturer. The specimens were heated at 5°C/min to 950°C without a holding period, followed by heating at 2°C/min to a final temperature of 1630°C. The final temperature was maintained for 120 min, after which the specimens were allowed to cool without active temperature regulation in the closed furnace.

The specimens were randomly divided into three groups (*n* = 10 per group) according to surface pretreatment:

- Airborne-particle abrasion (APA) reference group: The bonding surfaces were airborne-particle abraded with 110-μm aluminum oxide particles (Cobra Aluoxyd, Renfert GmbH, Hilzingen, Germany) for 10 s at a pressure of 2 bar, a distance of 10 mm, and perpendicular to the cementation surface [10].
- Silicon carbide bur (SiC bur) group: Before surface treatment, the zirconia specimens were used as manufactured, without additional polishing or pre-cleaning. The bonding surfaces were treated with a SiC Grinding Bur (Cimara, REF 1201, VOCO GmbH, Cuxhaven, Germany) operated in a low-speed handpiece at 7,000 rpm under dry conditions, without water irrigation and without additional hand pressure. These operating conditions followed the manufacturer’s instructions for obtaining a surface free from grinding residues and ceramic debris [11]. Ten strokes, consistent with the Cimara protocol described by Kirmali et al. [7], were applied over the entire bonding surface to standardize the treatment. A new bur was used for each specimen, as recommended by the manufacturer. Following treatment, grinding debris was removed under dry conditions using a short-bristled brush. All surface treatments were performed by the same operator.
- Airborne-particle abrasion followed by silicon carbide bur treatment (APA + SiC bur) group: Airborne-particle abrasion was performed first, followed immediately by the silicon carbide bur treatment described above.

The same surface-treatment protocol was applied to both the block-shaped and cylindrical zirconia components.

### Cementation procedure

**Figure 1:**
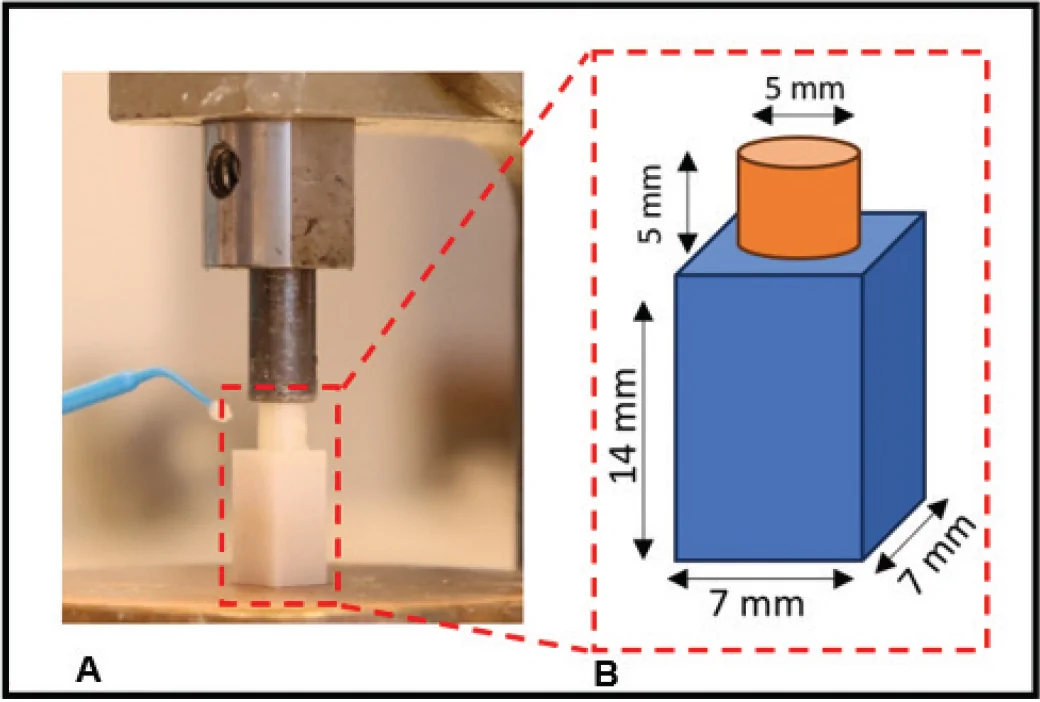
Image A illustrates the cementation process of the specimen components under a standardized pressure of 15 N. Image B presents a schematic illustration of a specimen along with its manufacturing dimensions.

A symmetrical zirconia–resin cement–zirconia configuration was deliberately selected to eliminate composite resin as a dissimilar adherend and an additional source of material-related variability. Each block-shaped specimen was cemented to a cylindrical counterpart from the same surface-treatment group (Figure 1). Clearfil Ceramic Primer Plus was applied to the bonding surfaces, followed by PANAVIA V5 adhesive resin cement (Kuraray Noritake Dental Inc., Okayama, Japan), in accordance with the manufacturer’s instructions. Light-curing was performed for 20 s from four directions at 90° angles using a curing light (D-Light Pro, GC Europe N.V., Leuven, Belgium) with an output intensity of 1,400 mW/cm².

The nominal bonding area was defined by the diameter of the cylindrical zirconia component. A diameter of 5.0 mm corresponded to a bonding area of 19.63 mm², calculated using A = πr², and this area was used to calculate the SBS. Excess cement extending beyond the perimeter of the cylindrical component was removed before light-curing. To standardize specimen seating, alignment, and cement extrusion, all specimens were assembled under a constant axial load of 15 N. The magnitude of the load was selected based on previously described zirconia bond-strength methodology using a comparable block-and-cylinder specimen configuration [10]. The load was maintained for 3 min to keep the components immobile during polymerization and to correspond with the manufacturer’s specified setting period for PANAVIA V5. The same cementation procedure was applied to all specimens and was performed by the same operator. The actual cement-layer thickness was not measured.

The cementation procedure was performed according to the manufacturers’ instructions, with the study-specific seating load and light-curing parameters described above. The artificial-aging procedure, SBS test, and failure-mode classification were adapted from previously described methods [9].

### Artificial aging

Artificial aging was performed using thermocycling (Thermocycler 1100/1200, SD Mechatronik GmbH, Feldkirchen-Westerham, Germany) for 5,000 cycles between 5 ± 2°C and 55 ± 2°C. Each cycle lasted 60 s, including a 10-s transfer time between baths. The primary outcome was SBS after artificial aging. All specimens were subjected to 5,000 thermocycles because the purpose was to compare the residual bond strength of the surface-treatment protocols after a standardized thermal challenge, rather than to quantify the effect of thermocycling itself [9, 12].

### Shear bond strength testing

**Figure 2:**
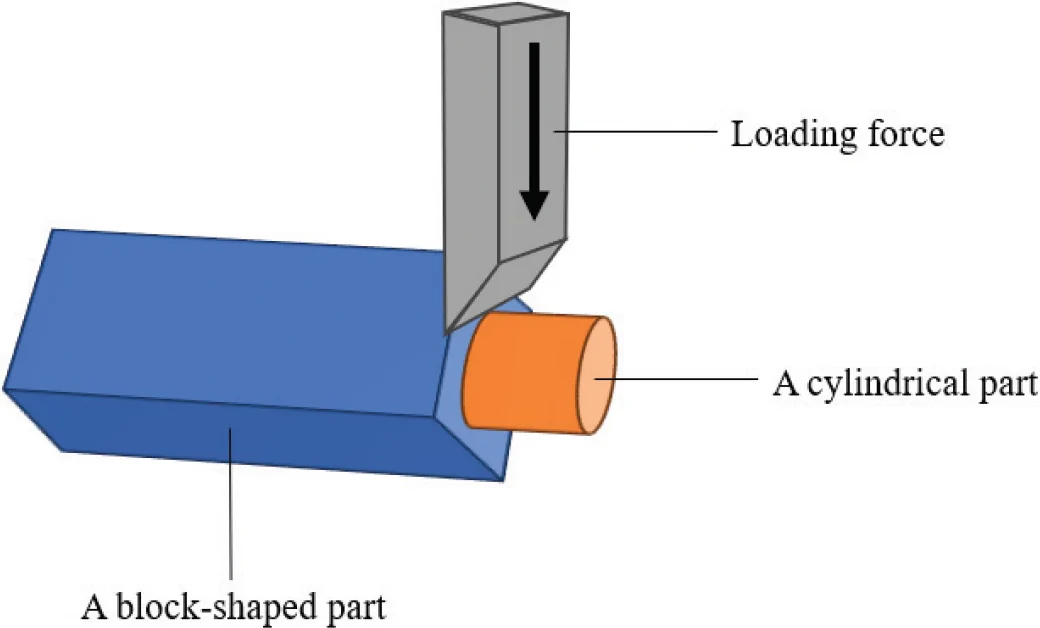
Schematic illustration of the shear bond strength test.

After thermocycling, the specimens were mounted in a universal testing machine (Instron 4465, Instron Corp., Canton, MA, USA) equipped with a knife-edged blade positioned perpendicular to the bonding interface (Figure 2). The crosshead speed was set to 1 mm/min, and loading was applied until failure occurred. SBS was calculated by dividing the maximum load at failure (N) by the bonding surface area (mm²), and the results were expressed in megapascals (MPa) [9].

### Failure mode analysis

Following debonding, the fracture surfaces were examined using a digital microscope (Dino-Lite, AnMo Electronics Corp., New Taipei City, Taiwan) at ×35 magnification. During the same examination, the zirconia fracture surfaces were inspected at ×35 magnification for visible cracks, chipping, or cohesive fracture of the zirconia.

Failure modes were classified according to the proportion of cement remnants on the bonding surface as follows:

- **Adhesive failure:** <10% of the bonding area covered with cement
- **Cohesive failure:** >90% of the bonding area covered with cement
- **Mixed failure:** 10%–90% of the bonding area covered with cement

### Statistical analysis

Statistical analyses were performed using IBM SPSS Statistics for Windows, Version 30.0 (IBM Corp., Armonk, NY, USA). Normality was assessed by visual inspection of normal Q–Q plots and by applying the Shapiro–Wilk test to the residuals from the one-way ANOVA model. Homogeneity of variances was assessed using Levene’s test. The primary analysis consisted of one-way ANOVA followed by Tukey’s post hoc test. Because a group-specific assessment indicated a deviation from normality in the SiC bur group, Welch’s ANOVA and the Kruskal–Wallis test were performed as sensitivity analyses. Pairwise Welch t-tests and Mann–Whitney U tests with Holm adjustment were used to evaluate whether the pairwise interpretation was affected. The magnitude of the overall effect of surface treatment was quantified using eta squared (η²) and omega squared (ω²). The significance level was set at α = 0.05.

## Results

**Figure 3:**
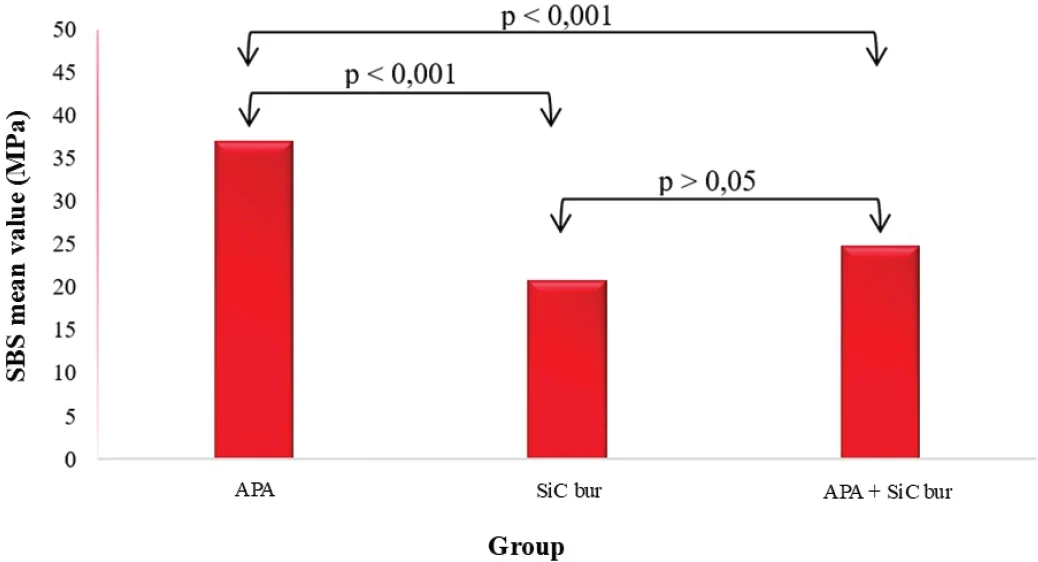
Mean shear bond strength (SBS; MPa) for each surface-treatment group and pairwise p-values. APA: airborne-particle abrasion; SiC bur: silicon carbide bur; APA + SiC bur: airborne-particle abrasion followed by silicon carbide bur treatment.

The analysis of the collected data showed that the APA reference group exhibited significantly higher mean SBS values than the SiC bur and APA + SiC bur groups (*p* < 0.001). The APA + SiC bur group showed a numerically higher mean SBS than the SiC bur group; however, the difference was not statistically significant (*p* > 0.05) (Figure 3). Table 1 presents the mean SBS values for each group, measured in MPa, along with the corresponding minimum and maximum values.

**Table 1:** Mean shear bond strength (MPa) for each group, including minimum and maximum values.

Group	*n*	Mean	Min.	Max.
APA	10	37.00	32.70	41.10
SiC bur	10	20.77	9.98	24.75
APA + SiC bur	10	24.86	18.28	33.56

APA: airborne-particle abrasion; SiC bur: silicon carbide bur; APA + SiC bur: airborne-particle abrasion followed by silicon carbide bur treatment.

The residuals from the one-way ANOVA model did not deviate significantly from normality (Shapiro–Wilk W = 0.978, *p* = 0.778), and homogeneity of variances was supported by Levene’s test (*F*(2,27) = 0.331, *p* = 0.721). A group-specific assessment indicated a deviation from normality in the SiC bur group (*W* = 0.749, *p* = 0.003), associated with one low observation. This observation was retained because no procedural or measurement error had been documented. Sensitivity analyses confirmed a significant overall effect of surface treatment using both Welch’s ANOVA (*F*(2,17.24) = 52.22, *p* < 0.001) and the Kruskal–Wallis test (H(2) = 19.96, *p* < 0.001). The pairwise sensitivity analyses yielded the same interpretation as the primary analysis: the APA reference group differed significantly from both the APA + SiC bur and SiC bur groups (Holm-adjusted *p* < 0.001), whereas the APA + SiC bur and SiC bur groups did not differ significantly (Holm-adjusted *p* ≥ 0.057). The overall effect of surface treatment was large (η² = 0.762; ω² = 0.737).

## Fracture analysis

**Figure 4:**
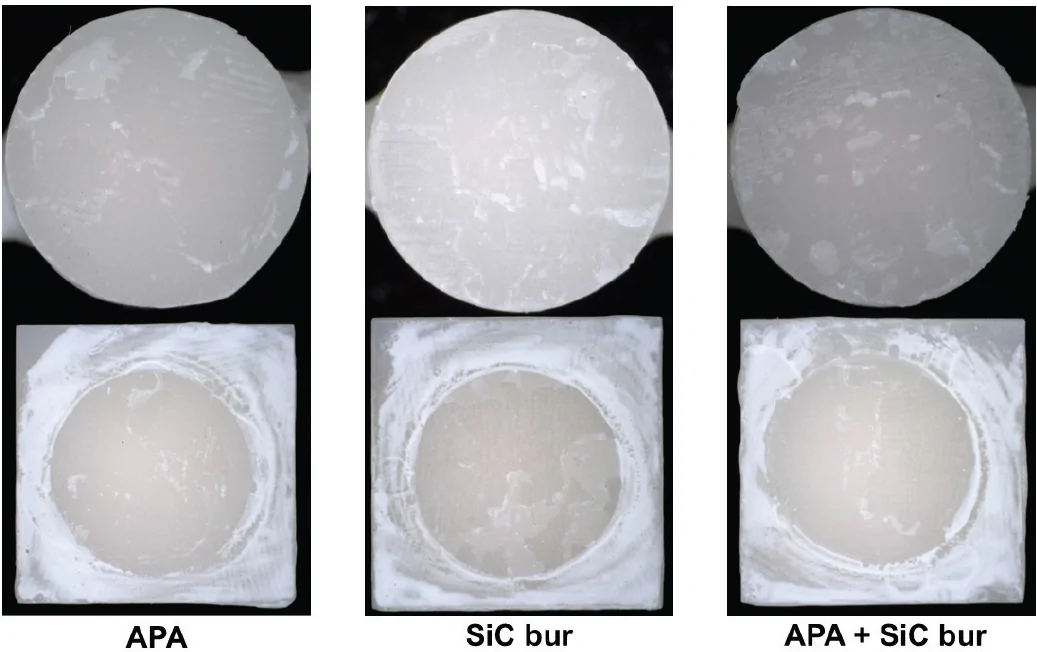
Representative microscopic images (×35 magnification) showing mixed fracture patterns based on cement distribution. APA: airborne-particle abrasion; SiC bur: silicon carbide bur; APA + SiC bur: airborne-particle abrasion followed by silicon carbide bur treatment.

All examined specimens exhibited mixed fractures. Residual cement was observed on the bonding surfaces of both specimen components, with coverage ranging from 10% to 90%, as illustrated in Figure 4. No visible cracks, chipping, or cohesive fracture of the zirconia was observed at ×35 magnification.

## Discussion

The findings of the present study revealed that the APA reference group exhibited significantly higher SBS values than the SiC bur and APA + SiC bur groups (*p* < 0.001), leading to rejection of the null hypothesis.

Airborne-particle abrasion was used as an active reference treatment because it is an established method for improving resin bonding to zirconia, and systematic evidence indicates that it generally increases bond strength compared with untreated zirconia [4, 6]. The specific airborne-particle abrasion parameters used in the present study -110-μm aluminum oxide particles, 2 bar pressure, 10-s application time, a distance of 10 mm, and perpendicular application – were based on a previously described zirconia-bonding protocol [10].

An untreated zirconia group was not included because the present study was designed to determine whether silicon carbide bur treatment could provide an alternative or an adjunct to airborne-particle abrasion, rather than to determine the absolute effect of either treatment compared with no surface treatment. Nevertheless, the absence of an untreated group is a limitation. Consequently, the present results cannot establish whether silicon carbide bur treatment increases bond strength compared with untreated zirconia.

The present findings differ from those of Janda et al., who reported high bond strength for a ceramic repair system that included the same silicon carbide grinding bur [9]. However, that study compared complete repair systems that differed in several components and application procedures and involved bonding composite resin to zirconia. Consequently, the specific contribution of the silicon carbide bur could not be isolated. In the present study, the airborne-particle abrasion reference group showed the highest SBS after aging. The APA + SiC bur group showed numerically higher SBS than the SiC bur group but significantly lower SBS than the APA reference group. One possible explanation is that the subsequent silicon carbide bur treatment altered the surface topography created by airborne-particle abrasion. However, surface roughness was not measured, and this interpretation therefore remains hypothetical.

Previous studies have specifically evaluated silicon carbide bur treatment for zirconia repair. Kirmali et al. found no significant difference between treatment with the Cimara silicon carbide grinding bur and CoJet regarding short-term repair bond strength [13]. Libecki et al. reported high initial bond strength after both air abrasion and silicon carbide bur treatment. After 150 days of water storage and 37,500 thermocycles, however, air abrasion produced more durable bond strength, whereas the silicon carbide bur group showed a significant reduction and the polished control specimens debonded spontaneously [14]. Taken together, these findings suggest that silicon carbide bur treatment may provide short-term bond strength comparable with particle abrasion in some repair protocols but may be less durable after aging. This pattern is consistent with the present post-aging results. Direct comparisons should nevertheless be made cautiously because the previous studies used composite repair configurations and different bonding systems and aging protocols. Furthermore, Kirmali et al. used CoJet with 30-μm silica-coated aluminum oxide particles, whereas the present study used 110-μm aluminum oxide particles and a 10-MDP-containing resin cement between two zirconia components.

Static bond-strength tests have limited ability to predict clinical performance [15, 16]. Therefore, the absolute SBS values obtained in the present study should be interpreted comparatively within this experimental model rather than against a proposed threshold for clinical acceptability.

One challenge encountered during the study was the handling of the small-sized specimen components, particularly the cylindrical components. The small dimensions complicated some procedural steps, especially when using the silicon carbide bur. For example, they made it difficult to maintain consistent control over the applied pressure and sweeping direction during surface treatment. To minimize the impact of these limitations on the results, each cylindrical specimen’s component was placed in a custom-manufactured plastic holder to facilitate handling during surface treatment. Furthermore, all experimental procedures were performed by the same operator in a single session to reduce variability. In addition, all steps were preceded by a pilot trial to ensure that each specimen underwent the treatment protocol under as standardized conditions as possible, thereby minimizing potential sources of error.

Notably, the fracture pattern observed in the current study was mixed. This contrasts with the findings of Kirmali et al., who reported solely adhesive failures [7], and with the outcomes of other studies where adhesive failure was dominant [10, 17]. For instance, Janda et al. evaluated the effect of five different repair systems on bond strength to zirconia and found that only the Ivoclar and VOCO systems produced mixed failures (13% and 53%, respectively), while the other systems resulted in fully adhesive failures [9]. The mixed fracture patterns in the present study may be attributed to the uniformity in specimen material and preparation: all specimens were made of zirconia, and all underwent identical surface treatments prior to cementation.

A limitation of this study was the difficulty in accurately determining the exact proportion of residual cement remaining on the surfaces, given the constraints of the methods and equipment used. Surface inspection could have been facilitated by the use of more advanced imaging techniques, such as scanning electron microscopy. Another contributing factor was the color similarity between the cement and zirconia, which further complicated visual differentiation. Nonetheless, the failure mode could still be classified based on the visible cement remnants, which clearly varied in quantity from 10% to 90%.

The zirconia specimens were examined only for macroscopically visible damage using a digital microscope at ×35 magnification. Because microscopic and subsurface defects were not characterized using methods such as scanning electron microscopy or micro-computed tomography, microcracks potentially induced by the surface treatments cannot be excluded.

A key consideration in the present study was the selection of a cement containing 10-MDP, given its well-documented efficacy in bonding to zirconia. Most systems today contain such a primer system. PANAVIA V5, a 10-MDP-containing resin cement, was chosen due to its widespread use. The cementation was carried out under a standardized load of 15 N to ensure consistent pressure across all specimens and to promote the uniform distribution of the cement layer between the bonded surfaces.

Although identical cementation conditions were used for all specimens, the actual cement-layer thickness was not measured and may have been influenced by treatment-induced differences in surface topography. This should be considered when interpreting the results.

To closely simulate the oral environment to which teeth and prosthetic restorations are exposed, thermocycling was employed as an artificial aging method. Çınar and Kırmalı demonstrated that there was no statistically significant difference between thermocycling with 3,000 or 6,000 cycles when assessing bond strength to zirconia [18]. Accordingly, the present study applied 5,000 thermocycles.

The evaluation after artificial aging was deliberately selected because immediate bond strength may overestimate the durability of resin bonding to zirconia, whereas thermal cycling challenges the bonded interface through repeated exposure to water and temperature-related dimensional changes [12, 19]. Since all groups underwent the same aging procedure, the results allow comparison of the surface-treatment protocols under identical post-aging conditions. However, because a non-aged reference group was not included, the magnitude of bond-strength degradation caused by thermocycling and any interaction between surface treatment and aging could not be determined. Future studies should include both immediate and aged measurements to investigate these effects.

Various laboratory methods are available for assessing bond strength, including tensile, microtensile, SBS, and microshear bond strength (μSBS) testing. SBS testing was selected because it is widely used in investigations of resin bonding to zirconia and facilitates comparison with previous studies [6, 20, 21]. However, measured bond-strength values depend on the test methodology and specimen configuration. Lopes et al. found that tensile, microtensile, shear, and microshear testing of thermocycled Y-TZP–resin cement interfaces produced method-dependent results. Increasing the bonded area was associated with lower bond-strength values, and shear tests may produce heterogeneous stress distributions at the adhesive interface [21]. The absolute SBS values obtained in the present study should therefore be interpreted within the specific experimental configuration and should not be directly compared with values obtained using different test methods [20, 21].

A limitation of this design is that the zirconia–resin cement–zirconia assembly contained two identically treated zirconia – cement interfaces rather than isolating a single zirconia – cement interface. Consequently, the measured failure load represents the strength of the complete assembly, and the absolute SBS values should be interpreted as comparative values within this experimental model rather than extrapolated directly to clinical restorations. Alternative SBS models using prefabricated composite resin cylinders isolate a single zirconia–cement interface and have been reported to provide reproducible testing conditions [20]. Future studies should therefore compare the present matched-zirconia configuration with composite resin, enamel, and dentin adherends under clinically relevant conditions [4].

This study aimed to investigate whether using the silicon carbide bur as a pretreatment could enhance reliability during the cementation of certain zirconia restorations that rely heavily on adhesive bonding, such as resin-bonded fixed partial dentures Maryland bridges. This study may be viewed as a preliminary step toward future investigations, including the exploration of alternative cement types and analysis of surface chemistry influence after silicon carbide bur on adhesion, which may further improve outcomes.

## Conclusion

Within the limitations of this in-vitro pilot study, silicon carbide bur treatment did not have a beneficial effect on SBS after 5,000 thermocycles. Airborne-particle abrasion with 110-μm aluminum oxide particles resulted in significantly higher SBS than silicon carbide bur treatment alone or airborne-particle abrasion followed by silicon carbide bur treatment. Thus, under the tested conditions, the silicon carbide bur cannot be recommended either as an alternative or as an adjunct to airborne-particle abrasion for improving the bond strength between zirconia and 10-MDP-containing resin cement.

### Clinical relevance

Existing recommendations for zirconia pretreatment remain unchanged. The additional use of a silicon carbide bur provided no beneficial effect and cannot be recommended as either an alternative or an adjunct to airborne-particle abrasion for improving zirconia–resin cement bonding.

## Data sharing statement

The data that support the findings of this study are available from the corresponding author upon reasonable request.
